# Supplementary material for: Monocytopenia, monocyte morphological anomalies and hyperinflammation characterise severe COVID‐19 in type 2 diabetes
Source: EMBO Mol Med. 2020 Sep 11;12(10):e13038. doi: 10.15252/emmm.202013038 (PMC7461002; doi:10.15252/emmm.202013038)
Supplement: Supplementary file 1 — Appendix [file EMMM-12-e13038-s001.pdf]

Appendix to Alzaid, Julla *et al*

"Monocytopenia, monocyte morphological anomalies and hyperinflammation characterise severe COVID-19 in type 2 diabetes"

Contents:

Page	Item
2	Appendix Table S1. Full blood count of COVID-19 patients in the non-diabetic (ND) and type-2 diabetic (T2D) groups at admission to hospital
3	Appendix Table S2. Flow cytometry-based counts of PBMCs, monocytes and monocyte subpopulations in non-diabetic (ND) and type-2 diabetic (T2D) patients with COVID-19
4	Appendix Table S3. Flow cytometry-based frequency of immune populations and subpopulations in non-diabetic (ND) and type-2 diabetic (T2D) patients with and without COVID-19
5	Appendix Table S4. Multivariate ANOVA (MANOVA) and univariate ANOVA F-values to test differences among cell-type percent according clinical characteristics
6	Appendix Table S5. Sample size and statistics table
7	Appendix Figure S1. Immunophenotyping approach and subpopulations quantified in peripheral venous blood.
9	Appendix Figure S2. Characterisation of monocyte morphology and inflammatory cells from peripheral circulation ND and T2D COVID-19 patients.
11	Appendix Figure S3. Characterisation of monocyte morphology and inflammatory cells from peripheral circulation ND and T2D COVID-19 patients.
13	Appendix Figure S4. Immunophenotypic analyses of T2D patients with COVID-19 treated in general wards or in ICU

Appendix Table S1. Full blood count of COVID-19 patients in the non-diabetic (ND) and type-2 diabetic (T2D) groups at admission to hospital

Cell counted (10^9/L or 10^6/mL)	ND (n=15)	T2D (n=30)	*p*-value	*Reference ranges (10^9/L or 10^6/mL)*
Leukocytes	6.3 (5.5-7.8)	6 (4.8-7.45)	0.255	*4.0 – 11.0*
Neutrophils	3.93 (3.23-5.27)	3.81 (2.92-5.08)	0.497	*2.0 - 7.5*
Lymphocytes	1.18 (1.07-1.75)	1.3 (0.91-1.65)	0.860	*1.5 - 4.5*
Monocytes	0.63 (0.48-0.76)	0.44 (0.33-0.62)	0.028	*0.2 - 0.8*

Appendix Table S2. Flow cytometry-based counts of PBMCs, monocytes and monocyte subpopulations in non-diabetic (ND) and type-2 diabetic (T2D) patients with COVID-19

Leukocyte populations (10^9/L or 10^6/mL)	ND (n=15)	T2D (n=30)	*p-value*
$CD45^+$ PBMCs	6.83 (3.71-13.44)	6.06 (2.35-15.49)	0,444
$CD14^+$ Monocytes	0.75 (0.24-1.71)	0.49 (0.13-1.26)	0,032
$CD14^{Hi}CD16^-$ Classical	0.55 (0.33-1.01)	0.35 (0.08-1.11)	0,005
$CD14^{Hi}CD16^+$ Intermediate	0.08 (0.01-0.32)	0.07 (0.002-0.25)	0,660
$CD14^{Lo}CD16^-$ Non-classical	0.02 (0.00-0.07)	0.01 (0.00-0.06)	0,054

Appendix Table S3. Flow cytometry-based frequency of immune populations and subpopulations in non-diabetic (ND) and type-2 diabetic (T2D) patients with and without COVID-19

	COVID-19		Uninfected	
Immune population (mean freq +/- SD)	ND (n=15)	T2D (n=30)	ND (n=36)	T2D (n=22)
Lymphocytes	10.8 +/- 6.1	12.2 +/- 10.1	22.2 +/- 9.5	31.1 +/- 18.4
CD8+	3.7 +/- 2.4	5.0 +/- 5.0	6.4 +/- 4.5	8.8 +/- 7.3
CD4+	6.8 +/- 5.1	6.1 +/- 4.4	10.4 +/- 5.0	13.5 +/- 15.0
DN	0.9 +/- 0.7	0.8 +/- 0.7	2.3 +/- 2.3	1.5 +/- 2.1
DP	0.1 +/- 0.1	0.07 +/- 0.07	0.2 +/- 0.1	7.3 +/- 10.1
Monocytes	9.9 +/- 2.0	7.6 +/- 3.6	9.0 +/- 2.2	9.9 +/- 4.9
Classical	7.9 +/- 3.6	5.7 +/- 2.5	7.4 +/- 2.7	8.3 +/- 6.7
intermediate	1.0 +/- 1.3	1.2 +/- 1.5	1.5 +/- 0.9	1.9 +/- 1.7
non-classical	0.3 +/- 0.3	0.2 +/- 0.2	0.4 +/- 0.3	0.2 +/- 0.4
DCs	1.5 +/- 0.9	1.7 +/- 0.9	0.9 +/- 0.4	1.9 +/- 2.1
mDCs	0.2 +/- 0.2	0.4 +/- 0.3	0.3 +/- 0.3	0.2 +/- 0.3
pDCs	0.3 +/- 0.5	0.2 +/- 0.3	0.3 +/- 0.2	0.3 +/- 0.5
B-cells	2.5 +/- 2.8	2.4 +/- 2.0	5.2 +/- 9.1	5.8 +/- 5.1
NK-cells	4.9 +/- 2.5	2.4 +/- 2.0	5.2 +/- 9.1	5.8 +/- 5.1
iNK cells	3.7 +/- 2.3	4.4 +/- 2.7	3.8 +/- 5.3	2.8 +/- 3.0
NKT- cells	1.1 +/- 1.3	1.7 +/- 1.7	0.9 +/- 0.9	1.4 +/- 1.5
Granulocytes	62 +/- 12.7	59.2 +/- 14.8	37.9 +/- 16.4	43.9 +/- 17.7

Appendix Table S4. Multivariate ANOVA (MANOVA) and univariate ANOVA F-values to test differences among cell-type percent according clinical characteristics

	MANOVA				ANOVA F-values			
	**DF**	**Pillai trace**	**Num DF**	**F**	**Classical**	**Non-Classi-cal**	**Intermediate**	**Lympho-cytes**
Diabetes	1	0,37	4,00	4.95**	6.43*	0,76	1,82	0,01
COVID-19	1	0,39	4,00	5.52**	9.65**	5.57*	0,12	4.21*
Sex	1	0,32	4,00	4.04**	0,30	0,82	1,05	13.03***
BMI	1	0,01	4,00	0,08	0,15	0,00	0,00	0,00
Hypertension	1	0,05	4,00	0,49	0,91	0,00	0,00	0,00

p-value * <0.05 ** <0.01 *** <0.001

Appendix Table S5. Sample size and statistical table

Figure	Panel	n	*p*-value
**1**	A	15 (ND) 30 (T2D)	*=0.0496
	B	15 (ND) 30 (T2D)	*=0,0170
	C	15 (ND) 30 (T2D) 30 (T2D non-COVID)	See appendix tables 1-3
**2**	A	15 (ND) 30 (T2D)	*= 0,0384; **=0,0082
	B	15 (ND) 30 (T2D)	p=0.0541; *=0,0478
	C	45 (15 ND + 30 T2D)	*=0,0482
	D	45 (15 ND + 30 T2D)	*=0.0317
	E	45 (15 ND + 30 T2D)	R=0.016 *p*= 0.01
	F	14 (ND) 28 (T2D)	**=0.0081; ***<0.0001
	G	11 (ND-Gen) 4 (ND-ICU) 19 (T2D-Gen) 11 (T2D-ICU)	*= 0,0186
**3**	A	14 (ND) 28 (T2D)	**=0,0081; *=0.0172; R2=0.11 *p*=0.03
	B	45 (15 ND + 30 T2D)	NA
	C	15 (ND) 30 (T2D)	NA
	D	45 (15 ND + 30 T2D)	NA
	E	15 (ND) 30 (T2D)	*=0.0329
	F	45 (15 ND + 30 T2D)	R2=0.07 *p*=0.04
	G	11 (ND-Gen) 4 (ND-ICU) 19 (T2D-Gen) 11 (T2D-ICU)	*p*=0.0154
	H	11 (ND-Gen) 4 (ND-ICU) 19 (T2D-Gen) 11 (T2D-ICU)	Classical [*=0.0154 amongst ND and 0.0267 amongst T2D]<br>Intermediate [*0.0236 between ND-Gen and T2D-ICU]
**4**	A	45 (15 ND + 30 T2D)	NA
	B	30 (19 Gen + 11 ICU)	*=0.037
	C	30 (19 Gen + 11 ICU)	Lymphocyte subpopulations *=0,039727<br>Monocyte subpopulations *=0,01147
	D	45 (15 ND + 30 T2D)	NA

NA=not applicable

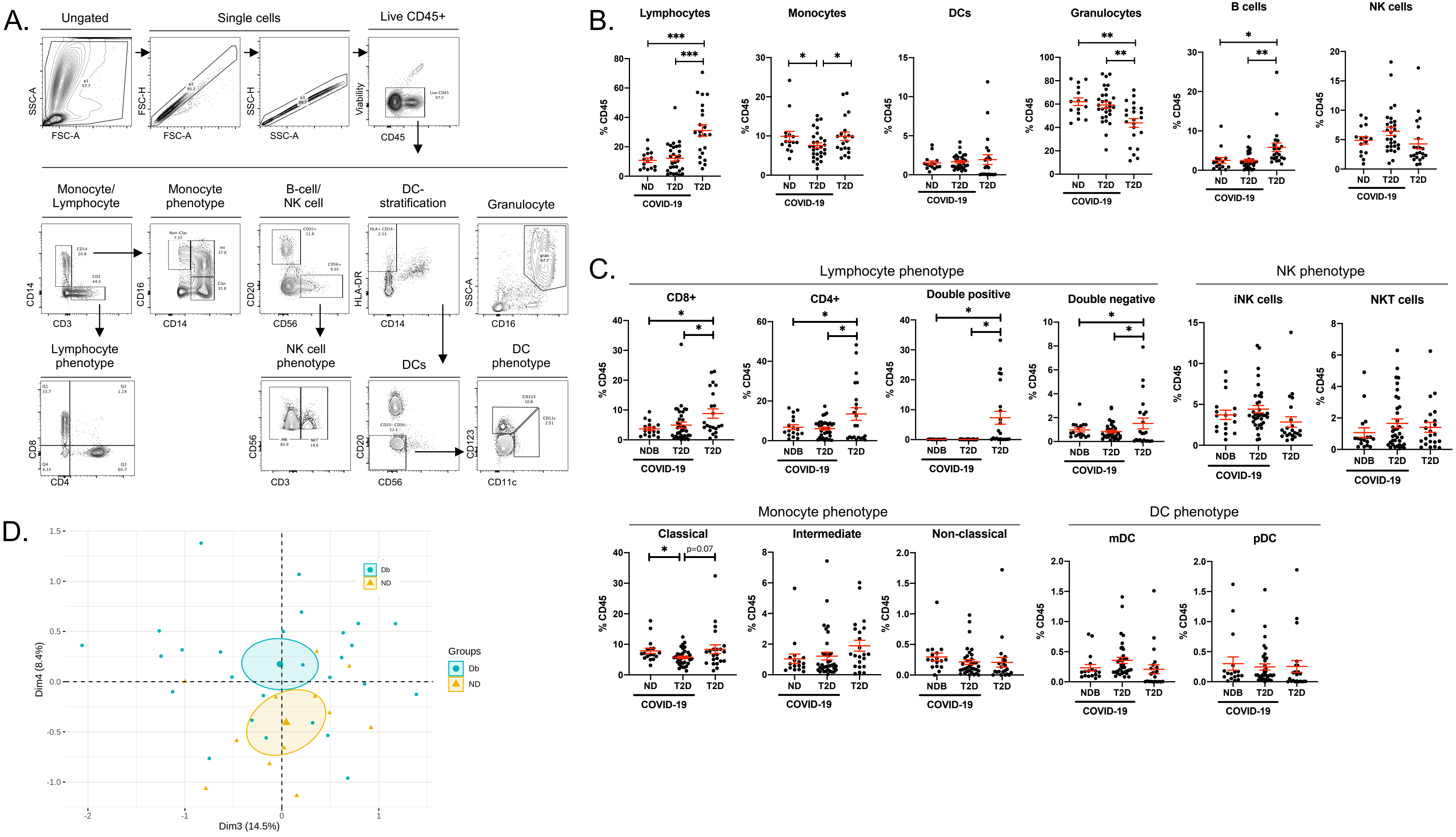

A.
Ungated
Single cells
Live CD45+
SSC-A
FSC-A
FSC-H
SSC-H
Viability
CD45
Monocyte/
Lymphocyte
Monocyte
phenotype
B-cell/
NK cell
DC-
stratification
Granulocyte
CD14
CD3
CD16
CD20
CD56
HLA-DR
Lymphocyte
phenotype
NK cell
phenotype
DCs
DC
phenotype
CD8
CD4
CD123
CD11c
B.
Lymphocytes
Monocytes
DCs
Granulocytes
B cells
NK cells
% CD45
ND
T2D
COVID-19
C.
Lymphocyte phenotype
NK phenotype
CD8+
CD4+
Double positive
Double negative
iNK cells
NKT cells
NDB
Monocyte phenotype
DC phenotype
Classical
Intermediate
Non-classical
mDC
pDC
p=0.07
D.
Dim4 (8.4%)
Dim3 (14.5%)
Groups
Db
ND

Appendix Figure S1. Immunophenotyping approach and subpopulations quantified in peripheral venous blood. Related to figure 1.

A. Markers and gating strategy applied for immunophenotypic analysis of immune populations in peripheral circulation

B. Major immune populations quantified in venous blood samples from type-2 diabetic (T2D) patients and in COVID-19 patients with T2D or in non-diabetic (ND) patients

C. Subpopulations quantified in peripheral circulation of T2D patients and of COVID-19 patients with T2D or ND

D. Principle component analysis on monocyte subpopulations from ND or T2D COVID-19 patients (d). Data are presented as mean +/- SEM

Data information: Differences between groups were evaluated with unpaired t-test. *p*-value. * : $p < 0.05$; ** : $p < 0.01$; *** : $p < 0.001$.

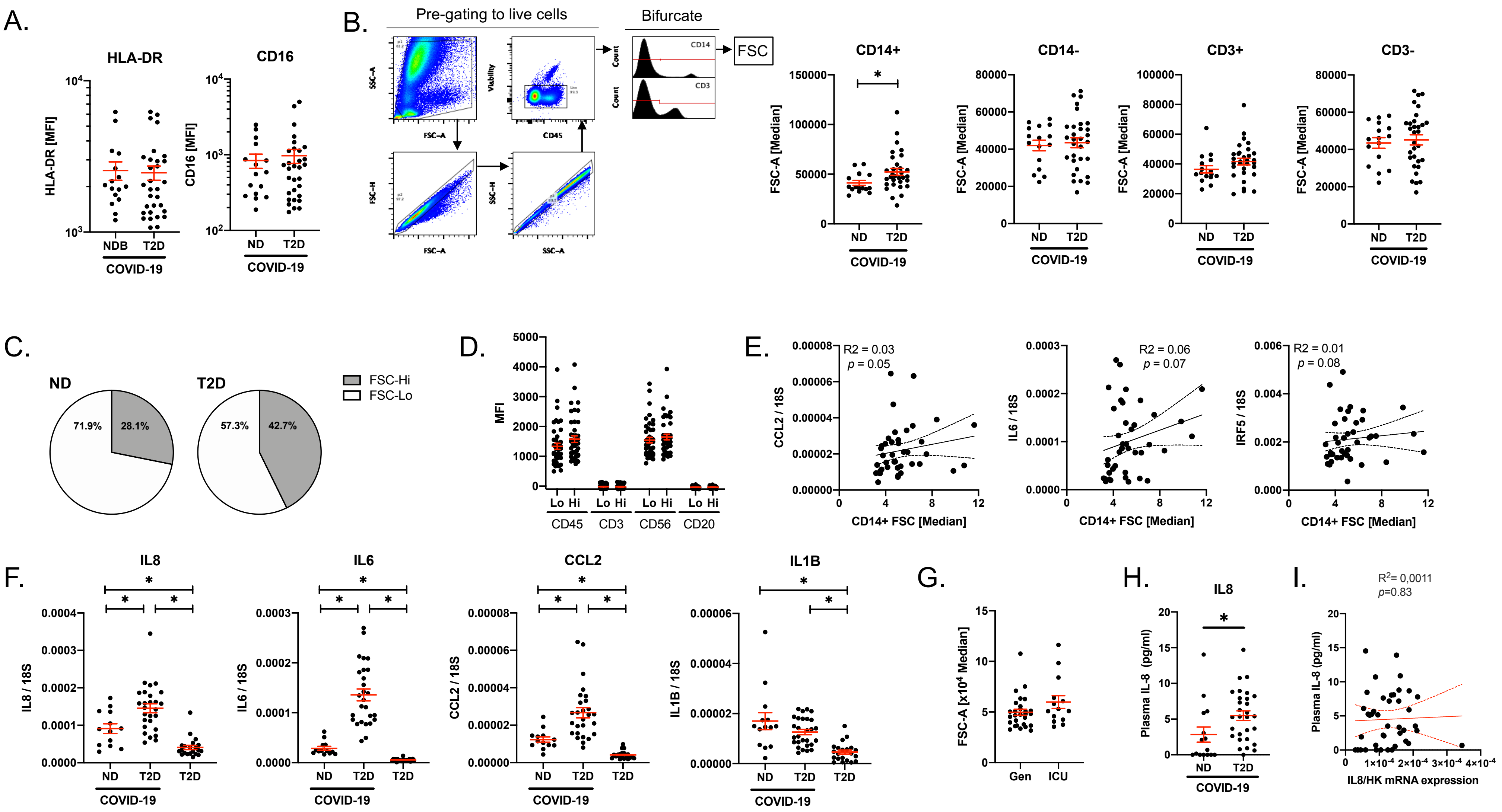
A.
HLA-DR
CD16
HLA-DR [MFI]
CD16 [MFI]
NDB
T2D
ND
COVID-19
B.
Pre-gating to live cells
Bifurcate
FSC
SSC-A
FSC-A
Viability
CD45
FSC-H
SSC-H
Count
CD14
CD3
CD14+
CD14-
CD3+
CD3-
FSC-A [Median]
C.
ND
T2D
FSC-Hi
FSC-Lo
71.9%
28.1%
57.3%
42.7%
D.
MFI
Lo Hi
CD45
CD3
CD56
CD20
E.
R2 = 0.03
p = 0.05
R2 = 0.06
p = 0.07
R2 = 0.01
p = 0.08
CCL2 / 18S
IL6 / 18S
IRF5 / 18S
CD14+ FSC [Median]
F.
IL8
IL6
CCL2
IL1B
IL8 / 18S
IL6 / 18S
CCL2 / 18S
IL1B / 18S
G.
FSC-A [x10^4 Median]
Gen
ICU
H.
IL8
Plasma IL-8 (pg/ml)
I.
R2= 0,0011
p=0.83
IL8/HK mRNA expression

Figure S2. Characterisation of monocyte morphology and inflammatory cells from peripheral circulation ND and T2D COVID-19 patients. Related to figure 2.

A. HLA-DR and CD16 expression in monocytes from ND and T2D COVID-19 patients.

B. Alternative bifurcate gating strategy to evaluate monocyte and lymphocyte size (FSC) in CD14 and CD3 +/- cells from ND and T2D COVID-19 patients.

C. Pie charts representing proportions of conventional (FSC^{Lo}) and large (FSC^{Hi}) monocytes in ND and T2D COVID-19 patients

D. Lineage marker expression in FSC^{Lo} and FSC^{Hi} monocytes from ND and T2D COVID-19 patients.

E. Correlation of inflammatory marker gene expression from peripheral blood mononuclear cells (PBMCs) to monocyte size (FSC) in ND and T2D COVID-19 patients

F. Gene expression of inflammatory markers in PBMCs from ND and T2D COVID-19 patients and from T2D patients without COVID-19

G. Monocyte size (FSC) in ND and T2D COVID-19 patients treated in general wards (Gen) or admitted to the intensive care unit (ICU).

H. Plasma levels of IL8 from ND and T2D COVID-19 patients

I. Correlative analyses of plasma IL8 and mRNA expression of IL8 in PBMCs from ND and T2D COVID-19 patients

Data information: Data are presented as mean +/- SEM. Differences between groups were evaluated with unpaired t-test (except for d. where pairing was by patient). Analyses of variance (ANOVA) or covariance (ANCOVA) were used for multiple group comparisons. For correlative analysis Spearman's test was carried out calculating a 2-tailed *p*-value. * : $p < 0.05$

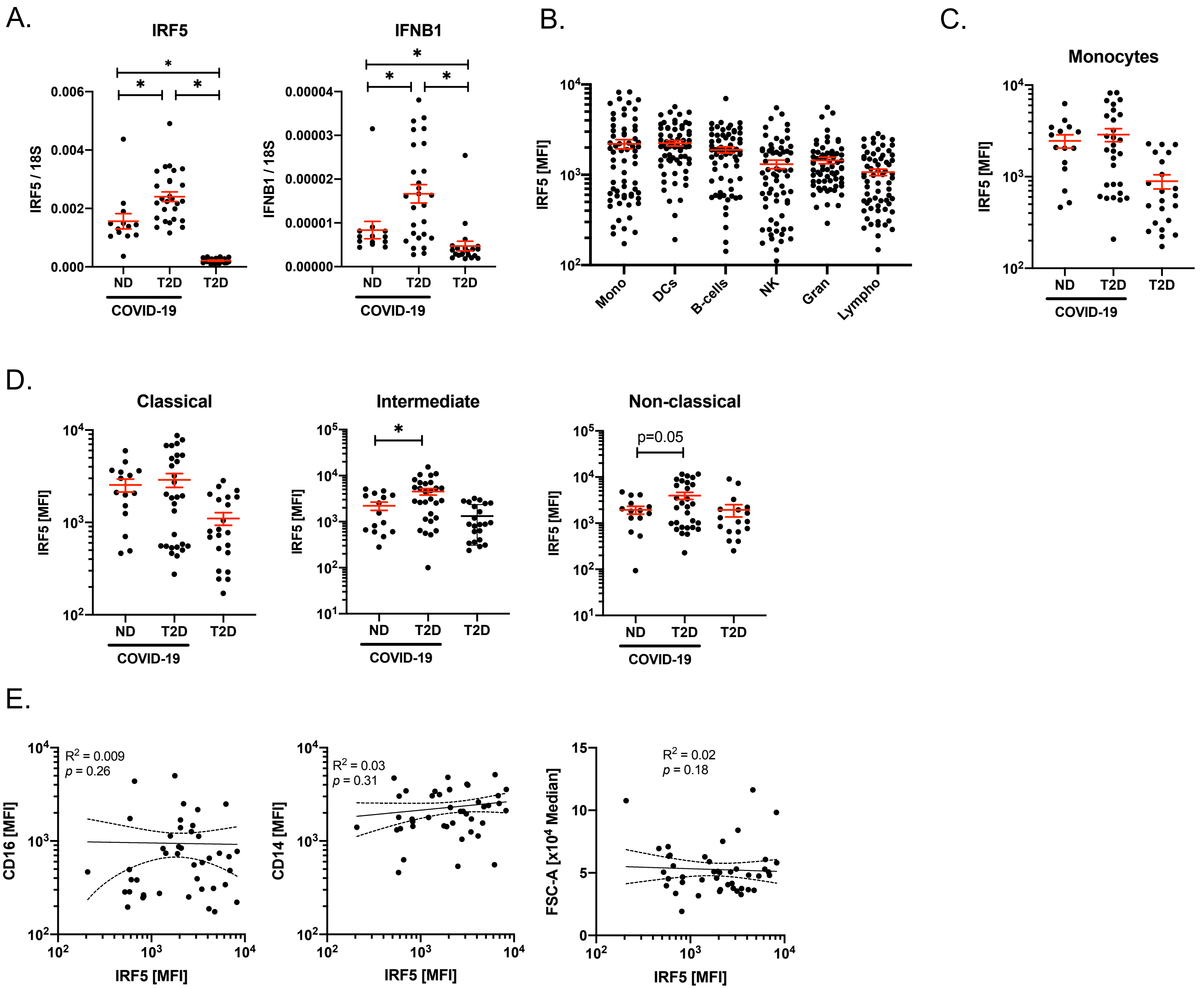
A.
IRF5
IFNB1
IRF5 / 18S
IFNB1 / 18S
ND
T2D
COVID-19
B.
IRF5 [MFI]
Mono
DCs
B-cells
NK
Gran
Lympho
C.
Monocytes
D.
Classical
Intermediate
Non-classical
p=0.05
E.
R^2 = 0.009
p = 0.26
R^2 = 0.03
p = 0.31
R^2 = 0.02
p = 0.18
CD16 [MFI]
CD14 [MFI]
FSC-A [x10^4 Median]
IRF5 [MFI]

Figure S3. Characterisation of monocyte morphology and inflammatory cells from peripheral circulation ND and T2D COVID-19 patients. Related to figure 3.

A. IRF5 and IFNB1 mRNA expression in COVID-19 patients with type-2 diabetes (T2D) and in non-diabetic (ND) patients.

B. IRF5 expression by flow cytometry in different immune cell populations found in circulation in ND and T2D COVID-19 patients.

C. IRF5 expression by flow cytometry in monocytes of ND and T2D COVID-19 patients and in T2D patients without COVID-19.

D. IRF5 expression by flow cytometry in monocyte subpopulations of ND and T2D COVID-19 patients and in T2D patients without COVID-19.

E. Correlative analyses of IRF5 expression by flow cytometry, to the expression of CD14, CD16 and to monocyte size (FSC) in monocytes of ND and T2D COVID-19 patients.

Data information: Differences between groups were evaluated with unpaired t-test. Analyses of variance (ANOVA) or covariance (ANCOVA) were used for multiple group comparisons. For correlative analysis Spearman's test was carried out calculating a 2-tailed *p*-value. * : $p < 0.05$

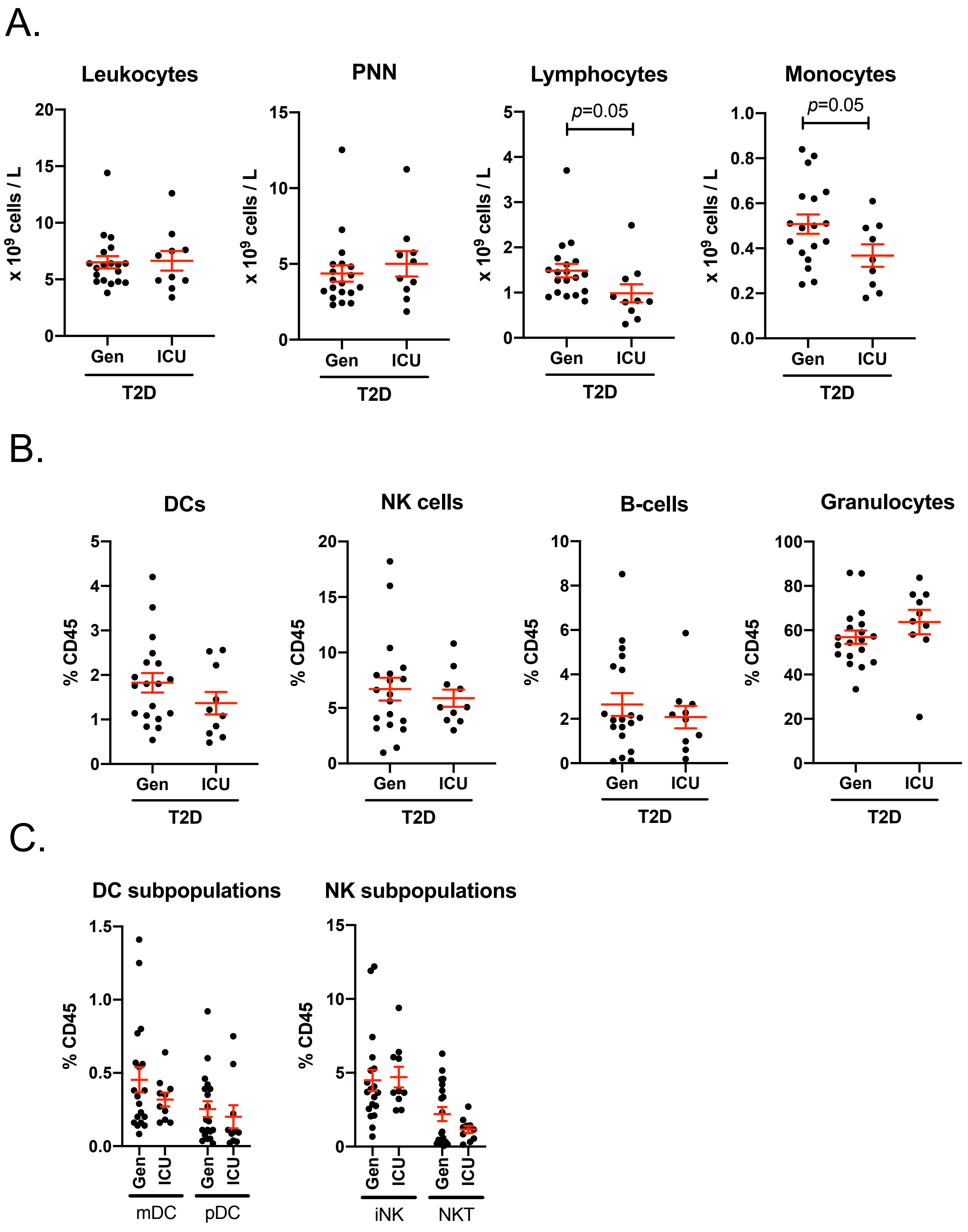
A.
Leukocytes
PNN
Lymphocytes
Monocytes
x 10^9 cells / L
p=0.05
p=0.05
Gen
ICU
T2D
B.
DCs
NK cells
B-cells
Granulocytes
% CD45
Gen
ICU
T2D
C.
DC subpopulations
NK subpopulations
% CD45
Gen
ICU
mDC
pDC
iNK
NKT

Figure S4. Immunophenotypic analyses of T2D patients with COVID-19 treated in general wards or in ICU. Related to figure 4.

A. Absolute quantification of circulating leukocytes from full blood counts at admission of type-2 diabetic (T2D) COVID-19 patients treated in general wards (Gen) or in the intensive care unit (ICU)

B. Flow cytometric quantification of dendritic cell (DC) natural killer (NK) cell, B cell and granulocyte frequency in peripheral blood of T2D COVID-19 patients treated in Gen or in the ICU

C. DC and NK subpopulation frequency in peripheral blood of T2D COVID-19 patients treated in Gen or in the ICU

Data information: Data are presented as mean +/- SEM. Differences between groups were evaluated with unpaired t-test.
